# Protective Effects of *Borago officinalis* (Borago) on Cold Restraint Stress-Induced Gastric Ulcers in Rats: A Pilot Study

**DOI:** 10.3389/fvets.2020.00427

**Published:** 2020-09-02

**Authors:** Alessandro Di Cerbo, Gianluca Carnevale, Rossella Avallone, Manuela Zavatti, Lorenzo Corsi

**Affiliations:** ^1^School of Biosciences and Veterinary Medicine, University of Camerino, Matelica, Italy; ^2^Surgical, Medical and Dental Department of Morphological Sciences Related to Transplant, Oncology and Regenerative Medicine, University of Modena and Reggio Emilia, Modena, Italy; ^3^Department of Life Sciences, University of Modena and Reggio Emilia, Modena, Italy

**Keywords:** *Borago officinalis*, stress-induced gastric ulcers, NMDA receptors, cAMP, anti-ulcer activity

## Abstract

Stress is a typical body's natural defense to a generic physical or psychic change. A specific linking mechanism between ulcer onset and psycho-physical stress prolonged exposure has been reported. We decided to investigate the possible effects of *Borago officinalis* L. (Borago) in preventing physical (stress)-induced gastric ulcers in a rat model. Eighty male Sprague-Dawley rats were randomly divided into 16 groups, pretreated with a control solution, omeprazole (20 mg/kg), Borago methanolic extract (25, 50, 100, 250, and 500 mg/kg), Borago organic extract (50, 100, 250, and 500 mg/kg), Borago aqueous extract (5, 10, 20, 30, and 40 mg/kg), and D(-)-2-Amino-5-phosphonovaleric acid (AP5) (25 mg/kg) and kept in stressful conditions such as water immersion and restraint-induced stress ulcers. The animals were sacrificed and their stomach scored for the severity and the number of gastric ulcers. Methanolic extract (500 mg/kg) significantly reduced both ulcer parameters (^***^p < 0.001 and ^**^p < 0.01, respectively). Aqueous and organic extract significantly decreased severity score at 5 and 10 mg/kg (^**^p < 0.01 and ^***^p < 0.001, respectively), and at 250 and 500 mg/kg (^***^p < 0.001), respectively, while gastric ulcers' resulted number significantly reduced only at 10 mg/kg (^*^p < 0.05) and at 500 mg/kg (^**^p < 0.01), respectively. On the other hand, aqueous extract significantly increased the mucosal gastric content of cAMP (^*^p < 0.05) and NR2A and NR2B subunits (^*^p < 0.05 and ^**^p < 0.01, respectively) at 5 mg/kg. Organic extract showed also a significant cytotoxic effect at 500 and 1,000 mg/kg with a 3T3 cell viability reduction of 43.6% (^**^p < 0.01) and 92.1% (^***^p < 0.001), respectively. Borago aqueous extract at 10 mg/kg could be considered as a potential protective agent against stress-induced ulcers, and it is reasonable to possibly ascribe such protective activity to a modulation of the NR2A and NR2B subunit expression.

## Introduction

Stress is a typical body's natural defense to a generic physical or psychic change, which involves a temporary alteration of organism homeostasis (1). Based on observations on gastric ulcer patients, a higher incidence of ulcerative disease was found among subjects who underwent a greater tension-emotional state. Stress frequency became definitely more assiduous during strong psychological tension periods (war or economic crisis) on a large portion of the population (2). Therefore, there seems to be a specific linking mechanism between ulcer onset and psycho-physical stress prolonged exposure. Thus, it is reasonable to affirm that psychosomatic factors, such as distress, play a pivotal role in the mechanisms involved in gastric ulcer induction (3).

Acute stress, besides an increased adrenocorticotropic hormone (ACTH) and corticosteroid release, is also characterized by an increase in catecholamine release, which, *per se*, has a protective effect at the gastric level. In fact, neurotransmitters such as noradrenaline (NE), dopamine (DA), and γ-aminobutyric acid (GABA) exert a protective effect on the gastric mucosa through an inhibitory control on acid secretion and motility. Such sympathetic system activation phenomenon represents, at the gastric level, a first phase defined as “resistance” during acute stress exposure. Due to the depletion of protective neuropeptides, e.g., neurotensin and β-endorphin, this phase is followed by an ulcerogenic phase, with the activation of the parasympathetic system characterized by ulcerogenic release factors such as acetylcholine and thyrotropic releasing hormone (TRH) and, as consequence, we assist to an increase in gastric motility and acid secretion. Acute stress-induced gastric ulcers reach their peak within 10–12 h following the stress stimulus but can rapidly regress after 24 h, if the stressful event does not repeat itself (4). Immunohistochemical studies carried out on gastric mucosae of rats and guinea pigs highlighted the presence of a glutamatergic neuron population in the ganglia of the myenteric and submucosal plexus (5, 6), and ultrastructural analyses revealed a glutamate concentration within small synaptic vesicles in the peripheral cholinergic axon terminals (7). Moreover, this latter study allowed the colocalization of acetylcholine and glutamate in the synaptic terminals of enteric neurons and thus hypothesizes their excitatory co-transmission in the enteric nervous system. Further studies highlighted the presence of N-methyl-D-aspartate (NMDA) receptor subunits NR1, NR2A, and NR2B mRNAs at myenteric and submucosal ganglia level (8, 9). This evidence, along with the ability of such receptors to increase intracellular calcium concentration, led to formulate several hypotheses concerning the involvement of the receptors in gastric ulcer onset (10). The demonstration that NMDA receptor stimulation with L-aspartic acid is able to regulate gastric secretion in rats by inhibiting histamine release supports the aforementioned hypothesis and strengthens the idea that a deregulation of such receptors might be relevant in the gastric mucosa-related pathologies (10, 11). Moreover, it is known that one mechanism involved in the gastric secretion is related to the increase in cyclic AMP (cAMP) through histaminergic H2 receptors (12). cAMP is a second messenger known to induce acid gastric secretion and pepsinogen, which are the two key factors involved in peptide ulcer onset (13). On the other hand, nitrogen monoxide stimulates mucus secretion and high cyclic GMP (cGMP) production from gastric mucosal cells, thus modulating some of the defensive factors of the stomach mucosa (14). Some studies suggested that the cAMP/cGMP ratio might be linked to the onset of ulcerative conditions (15, 16).

Recently, Gazwi and Mahmoud showed a protective effect of Borago against gastric ulcers induced by indomethacin. This effect was attributed to the antioxidant properties of the plant due to its content of polyphenols (17). *Borago officinalis* L. (Borago) is an annual herbaceous plant originating from the Mediterranean area and nowadays cultivated in the northern hemisphere but also in Australia, New Zealand, South Africa, and South America for its seed oil. In fact, Borago seed oil is rich in γ-linolenic acid. This plant, traditionally used for its antispasmodic (18), antipyretic (19), antihypertensive (20), aphrodisiac (19), and diuretic (21) properties, has been also considered useful in the treatment of asthma (18), bronchitis (19), cramps (19), diarrhea (22), palpitations (19), and kidney pain (23, 24). As for Borago constituents, tannins, resins, ascorbic acid, niacin, riboflavin, thiamine, silicic acid, flavonoids, coumarins, and several polyphenolic (caffeic, rosmarinic, and chlorogenic acid) and pyrrolizidine alkaloid compounds have been detected (18, 25, 26). Based on recent observations on indomethacin-induced ulcers (17), we decided to investigate the possible effects of Borago in preventing physical (stress)-induced gastric ulcers in a rat model.

## Materials and Methods

### Methanolic Extract Preparation

Borago leaves (100 g) were purchased from Maitex International S.a.s [Marina di Campo (LI), Italy], crumbled and macerated in 1 L of methanol for 24 h to obtain the hydro-alcoholic crude extract. The latter was then filtered, evaporated, lyophilized, titrated in 1.5% rosmarinic acid, and used for all the experiments described below.

### Alkaloid Fraction Isolation

Methanolic extract was resuspended in a 100 ml solution of HCl 2N acidulated water (pH between 2 and 3) and ethyl acetate (50:50). The mixture was introduced into a separating funnel and vigorously shaken. The funnel was left vertically until aqueous and organic phase separation. Then, the organic phase was collected while the aqueous one was newly resuspended with 50 ml of ethyl acetate and introduced into a separating funnel. Once both phases were separated, the aqueous one was frozen and then lyophilized, while the organic phase was evaporated by means of a rotating evaporator. The organic phase was suspended with methanol and water (50:50), frozen and lyophilized.

### Animals

Eighty male Sprague-Dawley rats (235–370 g) were purchased from Charles River Laboratories S.p.A (Calco, Italy). All animals were housed in metabolic cages with 12:12 h light/dark cycles at 22°C and 60% of humidity and given food and water *ad libitum* for 15 days. Rats were randomly assigned to different experimental groups (five for each group) and kept fasted for 24 h before each experiment. Each treatment was performed by gavage administration using a volume of 5 ml/kg.

### Stress-Induced Gastric Ulcer Model and Treatment

Animals kept fasted for 24 h were grouped as shown:

- group 1 (*n* = 5) was given a control solution of water + 5% tween 80 (control),
- groups 2–6 (*n* = 5, each) were administered with a Borago methanolic extract (dissolved in the control solution) at a dosage of 25, 50, 100, 250, and 500 mg/kg, respectively
- groups 7–10 (*n* = 5, each) were administered with a Borago organic extract (dissolved in the control solution) at a dosage of 50, 100, 250, and 500 mg/kg, respectively
- groups 11–14 (*n* = 5, each) were administered with a Borago aqueous extract (dissolved in the control solution) at a dosage of 5, 10, 20, and 40 mg/kg, respectively
- group 15 (*n* = 5) was given a D(-)-2-Amino-5-phosphonovaleric acid (AP5) (dissolved in the control solution) at a dosage of 25 mg/kg
- group 16 (*n* = 5, each) were administered with an omeprazole (dissolved in the control solution) at a dosage of 20 mg/kg.

The concentration of the aforementioned extracts was chosen based on previous papers (17, 27–29).

Immediately after the last solution administration, animals were placed into plexiglass restraint cages without any movement possibility as described by Gurbuz and Yesilada (30). Cages were placed into the water at 19–21°C so that the rats were dipped until the xiphoid process. The animals were kept in this stressful condition for 7 h and then sacrificed to collect their stomach. Then, each stomach was dissected following the great curvature and washed accurately with a saline. Macroscopic (Nikon stereomicroscope, SMZ1) analyses were conducted to assess ulcers' number and intensity. Then the mucosa was separated from each stomach and stored at −80°C in liquid nitrogen for further evaluations. The number and intensity of gastric ulcers were independently rated by two blinded assessors using a numerical rating scale with 6 degrees of severity (0 = normal mucosa; 1 = reddened mucosa or with a limited number of petechiae; 2 = mucosa erosion or several petechiae or small ulcers with a diameter <1 mm; 3 = up to three large ulcers with a diameter >1 mm; 4 = more than three large ulcers with a diameter >1 mm; 5 = perforated ulcer) (31).

### cAMP, cGMP, and Nitric Oxide Determination

Cyclic AMP EIA Kit, Cyclic GMP kit, and Nitrate/Nitrite Colorimetric Assay Kit (Cayman, USA) were used to determine cAMP, cGMP, and NO, respectively, in gastric tissue extracts from stress-induced ulcer rats following the manufacturer instructions.

### Reverse Transcription Polymerase Chain Reaction

Portion of stomach samples undergoing RNA extraction were homogenized and re-suspended in extraction buffer with Qiagen total RNA purification kits (Qiagen, Valencia, CA, USA), following the manufacturer instructions. Total RNA was quantified using a NanoDrop 2000 spectrophotometer (Thermo Fisher Scientific, Waltham, MA, USA), and 100 ng was used to synthesize cDNA with the High Capacity cDNA Retro-transcription Kit (Invitrogen). Quantitative real-time PCR (qRT-PCR) was performed using an ABI PRISM 7900 detection system (Applied Biosystems). All probes and primers used for mRNA amplification were designed by Applied Biosystems. In particular, for NR1 NM_017010.1 (Forw.: 5′-GCTTTTGCAGCCGTGAAC-3′; Rev.: 5′GGGCTCTGCTCTACCACTCTT-3′); for NR2A NM_012573.3 (Forw.: 5′-GACAACAGTGGACAACAGCTTTG-3′; Rev.: 5′-GAAGGAGGTGTCCAGTGTGATC-3′); for NR2B NM_012574 (Forw.: 5′-ATGCTCAACATCATGGAAGAATATGACT-3′; Rev.:5′CTGCGGATCTTGTTCACAAAGTC-3′) and for NR2D NM_022797.1 (Forw.: 5′ -AGTACGACTGGACATCCTTTGTG-3′; Rev.:5′-AGCACCTCGATGTATGACAAGAAG-3′). Each cDNA sample was run in triplicate using the Taqman Universal PCR Master Mix (Invitrogen), and glyceraldehyde-3-phosphate dehydrogenase (GAPDH) was used as an endogenous control. Quantification of RT-PCR signals was performed using the 2^−ΔΔct^ relative method. This procedure calculates the relative changes in gene expression of the target gene normalized with an endogenous control and compared with a calibrator sample.

### Cell Culture

BALB/3T3 clone A31-1-1 cells were provided by the Istituto Zooprofilattico Sperimentale (IZSBS, Brescia, Italy). The de-frozen cell culture assays were always maintained in a sub-confluent state (<80% of confluence). Cells were cultured using Dulbecco's Modified Eagle's Medium (DMEM) supplemented with 10% FBS (Australian origin) and 0.5% (v/V) Penicillin-Streptomycin(Invitrogen, Italy).

### Cell Viability

BALB/3T3 cells were plated on 96-well plates (Euroclone, Italy) at a concentration of 30,000 cells/cm^2^. After exposure to desired concentrations of Borago extracts, 20 μl MTS (CellTiter 96^®^ Aqueous One Solution Cell Proliferation Assay, Promega, Italy) was added to each well and incubated for a period of 2.5 h. Finally, absorption was measured at 492 nm using a Multiscan microplate reader (Labsystem Multiscan^®^ MCC/340, Finland).

### Statistical Analysis

Data were analyzed using GraphPad Prism 8 software (GraphPad Software, Inc., La Jolla, CA, USA). All data are presented as means ± standard error of the mean. Differences in number and intensity of gastric ulcers, mRNA expression of NMDA receptor subunits, cAMP, cGMP, and NO concentration were analyzed using a one-way analysis of variance (ANOVA) followed by Tukey's multiple comparisons test vs Ctrls. A ^*^p < 0.05 was considered significant.

## Results

### Effect of Borago Extract Administration on Stress-Induced Ulcers

**Figure 1:**
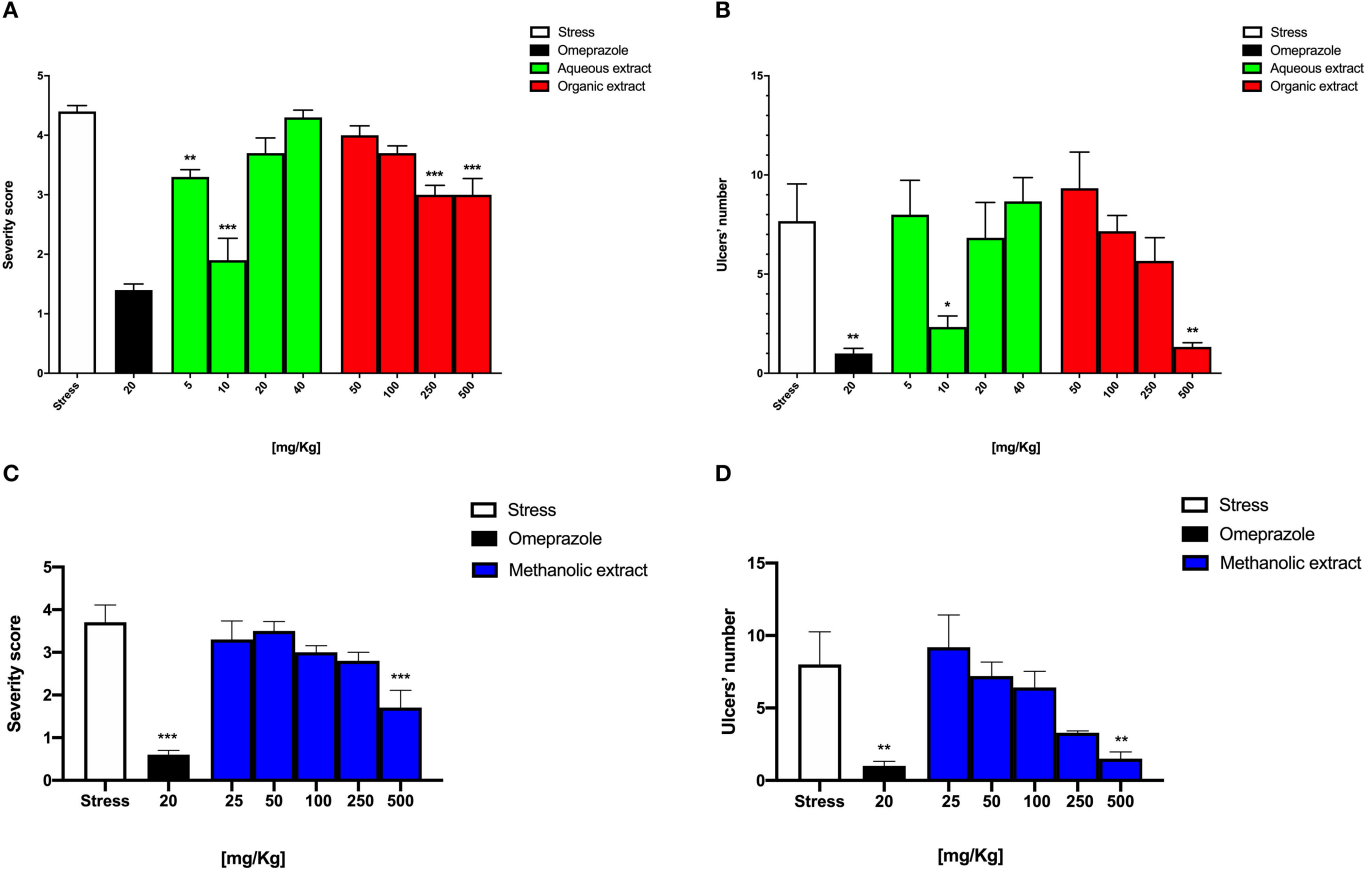
Graphical representation of Borago methanolic extract effect at different dosages on **(A)** intensity and **(B)** number of stress-induced gastric ulcers and Borago organic and aqueous extract effect at different dosages on **(C)** intensity and **(D)** number of stress-induced gastric ulcers.

**Figure 2:**
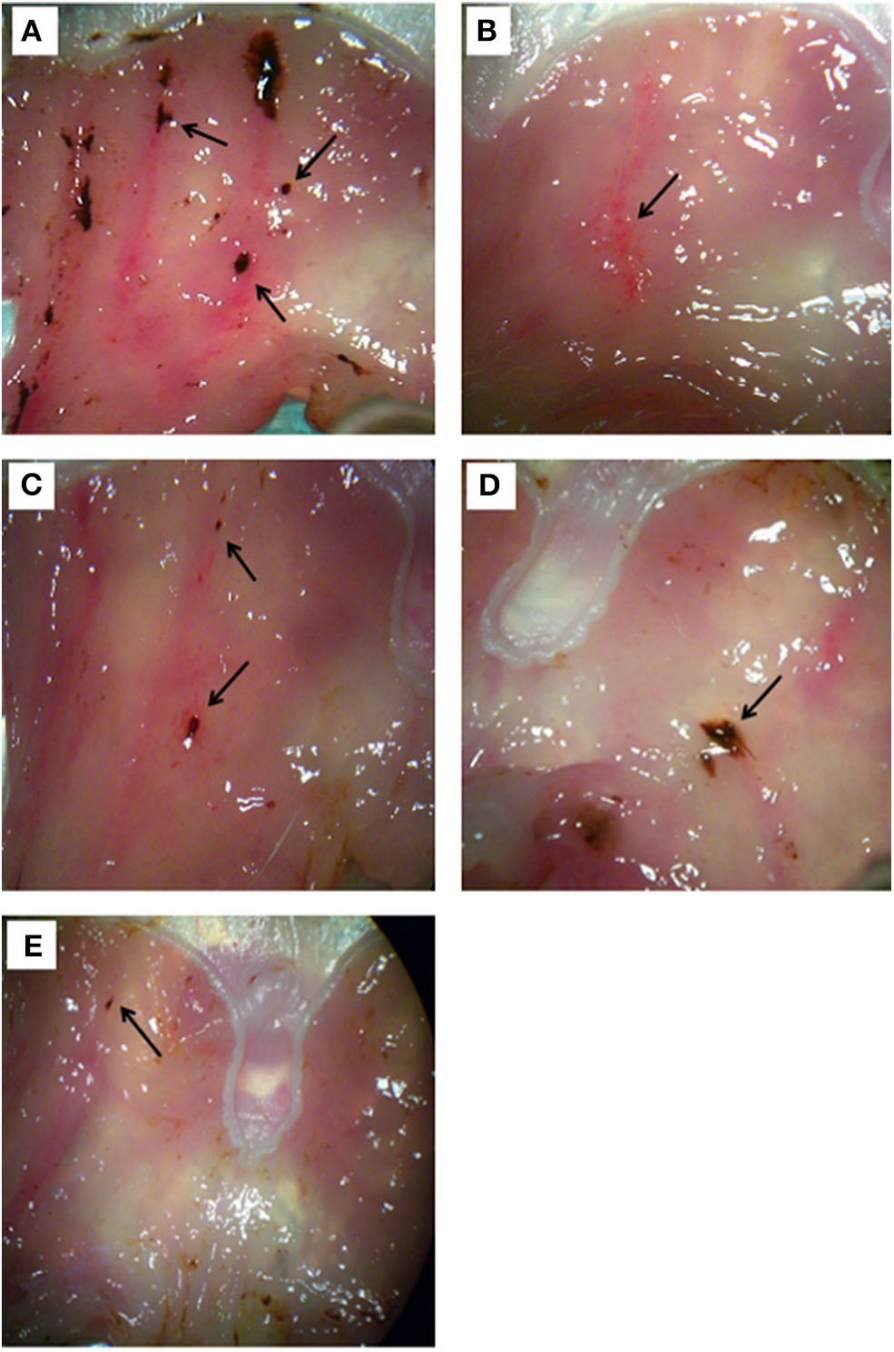
Morphological examination of gastric tissue from rats treated with **(A)** stress stimulus, **(B)** omeprazole 20 mg/kg, **(C)** Borago aqueous (10 mg/kg), **(D)** organic (500 mg/kg), and **(E)** methanolic extract (500 mg/kg). The ulcers are indicated by the arrow.

The stress-induced gastric ulcer model showed a high severity score and gastric ulcer number, 3.7 ± 0.4 and 8.0 ± 2.2, respectively, while an opposite significant trend was observed for omeprazole, 0.6 ± 0.1 (^***^p < 0.001) and 1.0 ± 0.3 (^***^p < 0.001), respectively (Figure 1) as also shown (Figures 2A,B). Conversely, Borago methanolic extract administration seemed to reduce both the severity score and the gastric ulcer number in a dose-dependent manner. However, only the pretreatment with 500 mg/kg significantly reduced both ulcer parameters to 1.7 ± 0.4 (^***^p < 0.001) and 1.5 ± 0.5 (^**^p < 0.01), respectively (Figures 1C,D).

In order to determine the main component of the Borago extract responsible for the antiulcer activity, a separation of pyrazolidine components was performed. We therefore proceeded to isolate the alkaloid fraction from the methanolic extract with the obtainment of an organic and aqueous extract, with the latter possibly containing the alkaloids.

As reported in Figure 1A, the severity score of the Borago aqueous and organic extract significantly decreased at 5 and 10 mg/kg to 3.3 ± 0.1 (^**^p < 0.01) and 1.9 ± 0.4 (^***^p < 0.001), respectively, and at 250 and 500 mg/kg to 3.0 ± 0.1 (^***^p < 0.001) and 3.0 ± 0.3 (^***^p < 0.001), respectively. Conversely, the gastric ulcer number significantly reduced only at 10 mg/kg to 2.0 ± 0.5 (^*^p < 0.05), and at 500 mg/kg to 0.7 ± 0.1 (^**^p < 0.01) for aqueous and organic extract, respectively (Figure 1B).

As mentioned above, macroscopic examination of gastric ulcers showed that the omeprazole group had almost no ulcer spots (Figure 2B) with respect to the stress group (Figure 2A). On the other hand, the Borago aqueous (10 mg/kg), organic and methanolic (500 mg/kg) extract showed some minor ulcer spots (Figures 2C–E).

**Figure 3:**
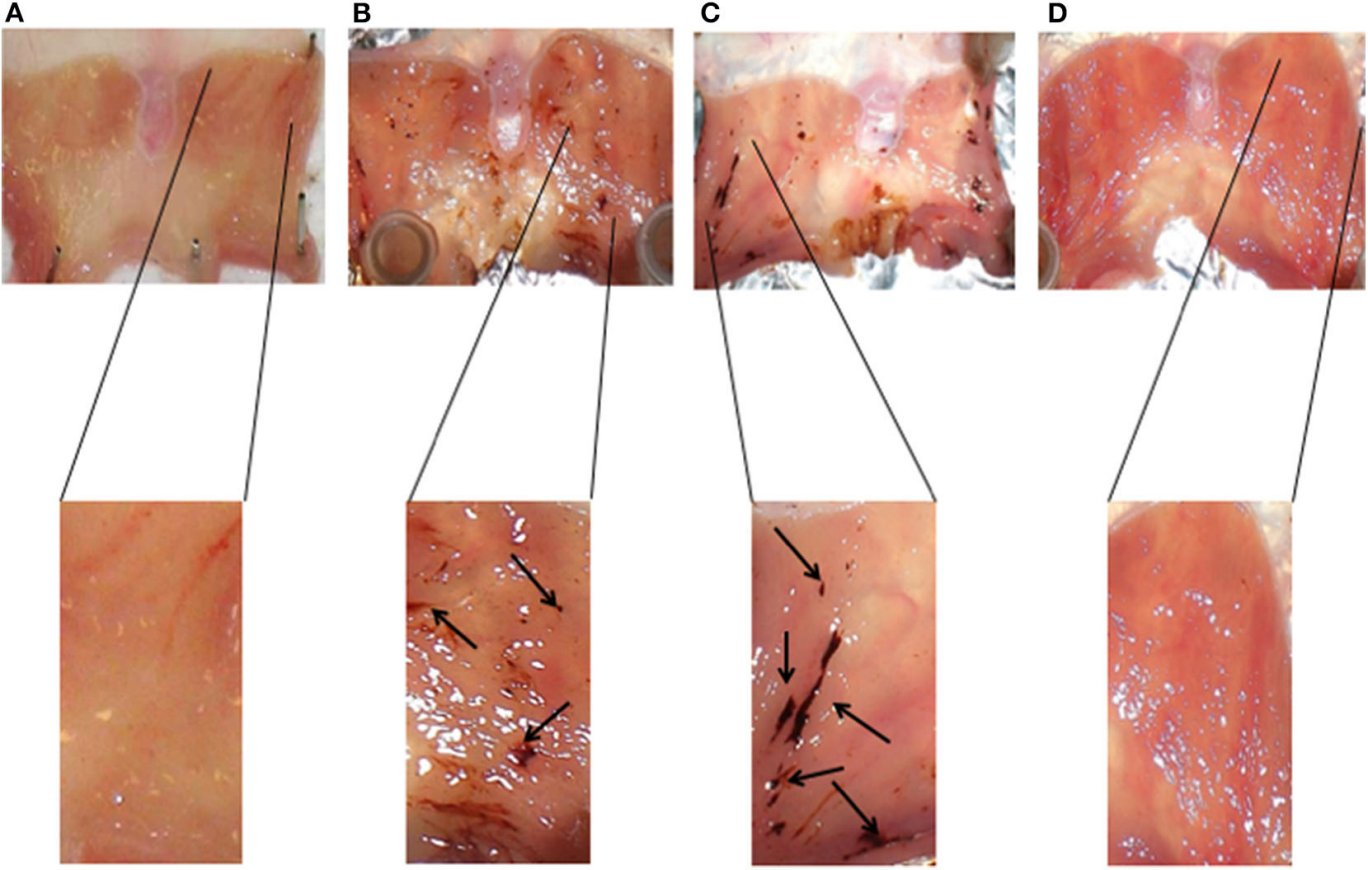
Representative histology of gastric mucosa samples of animals subjected to different experimental conditions: non-stressed rats **(A)**, stressed rats **(B)**, stressed rats pretreated with AP5 **(C)**, and stressed rats pretreated with omeprazole **(D)**.

Interestingly, the results obtained on the rats subjected to stress (Figure 3B) and pretreated with AP5 (Figure 3C), a selective antagonist of the NMDA receptor system, showed a worsening of the pathophysiological conditions of the gastric mucosa compared to the non-stressed animals (Figure 3A). Indeed the ulcerative lesions were more extensive and deeper.

### cAMP, cGMP, and NO Determination

**Figure 4:**
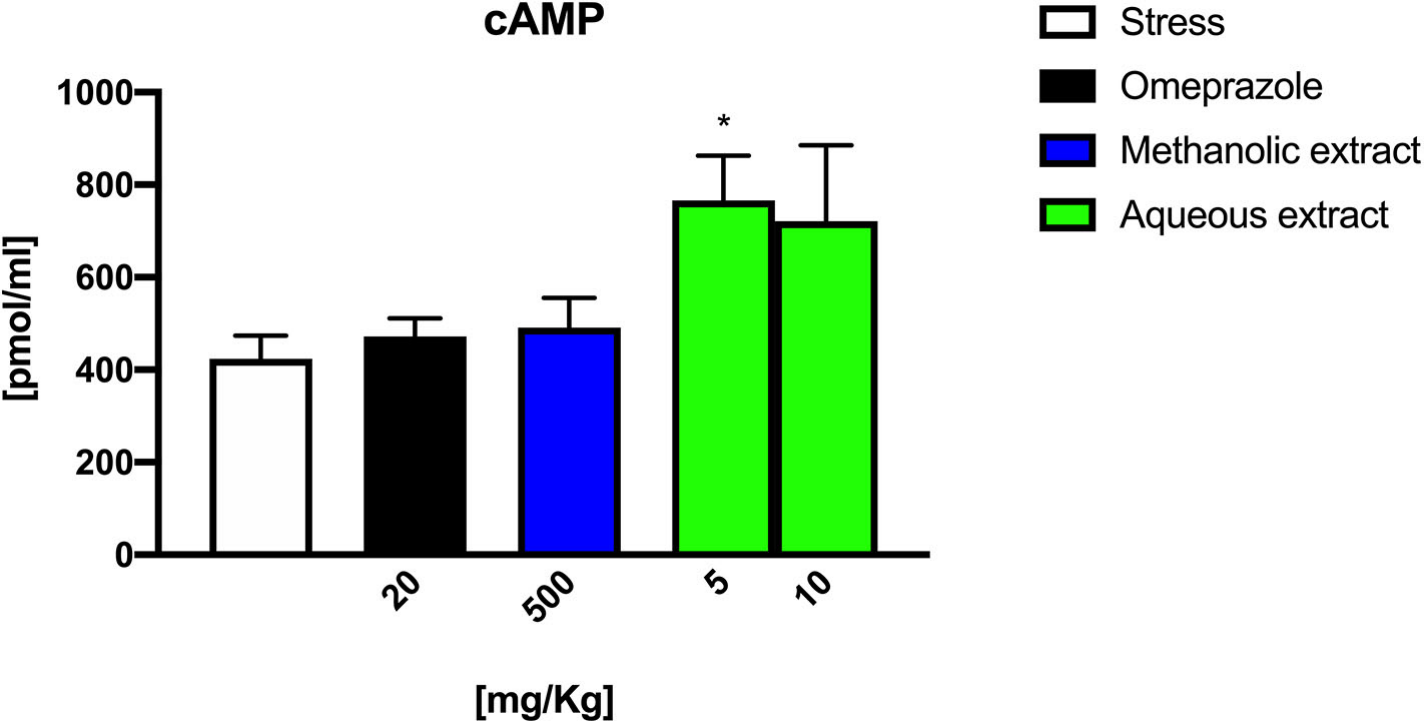
cAMP levels in gastric tissue extracts from stress-induced ulcer.

cAMP and cGMP levels were measured in gastric tissue extracts from stress-induced ulcer rats to verify whether the Borago gastro-protective mechanism might be due to a modulation of cyclic nucleotide levels. In Figure 4, the cAMP levels measured in gastric tissue extracts from stress-induced ulcer rats are presented.

The results showed that the Borago methanolic extract and omeprazole did not alter the amount of cAMP. On the other hand, the Borago aqueous extract significantly increased the mucosal gastric content of cAMP at 5 mg/kg (^*^p < 0.05), while the increase observed at 10 mg/kg was not significant. The data obtained on both cGMP and NO content were barely detectable, and they did not show any difference at all times tested (data not shown).

### Effect of Extracted Fractions on Glutamate Receptors in Stress-Induced Ulcer Model

**Figure 5:**
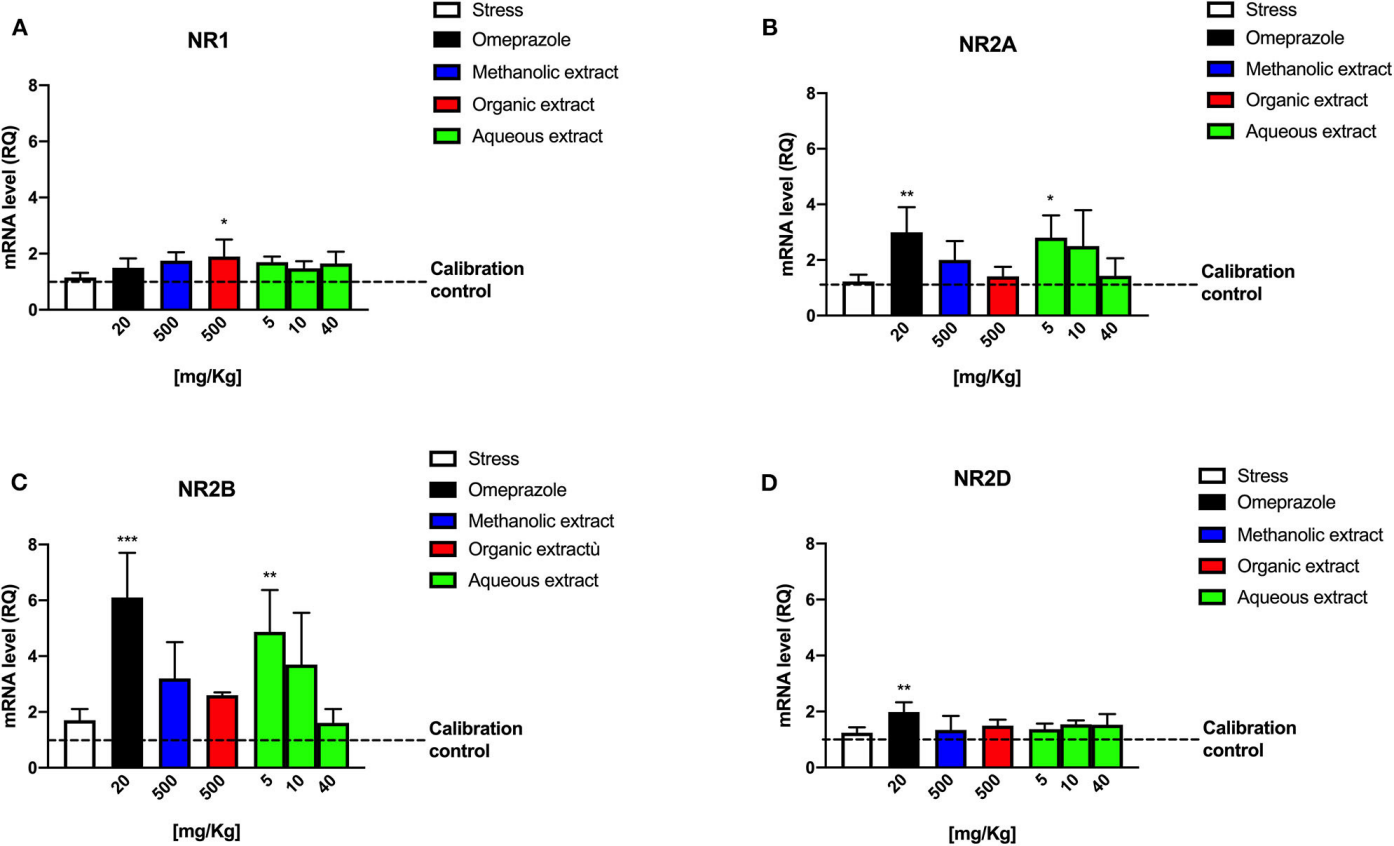
mRNA levels of NMDA subunits in gastric tissue extracts from stress-induced ulcer.

We decided to investigate the possible role of the Borago extract in modulating glutamate receptor expression due to their key role in the pathophysiologic response to ulcer-inducing agents. In order to verify any possible molecular modulation of the glutamatergic NMDA receptor, we evaluated mRNA expression of its different subunits by means of real-time reverse transcription-polymerase chain reaction (RT-PCR) on stomach tissue samples from a stress-induced ulcer rat model treated with omeprazole and Borago extracts. RT-PCR analysis revealed that all mRNAs of NMDA subunits were expressed, but all treatments modified their expression pattern (Figure 5).

Noteworthy is the significant expression of NR2A and NR2B subunits following Borago aqueous extract (5 mg/kg, ^*^p < 0.05 and ^**^p < 0.01, respectively) and omeprazole administration (Figures 5B,C, ^**^p < 0.01 and ^***^p < 0.001, respectively), the significant expression of NR2D subunit expression following omeprazole administration (Figure 5D, ^**^p < 0.01), and the lack of any significant effect exerted by the stress stimulus. No significant variations of genic expression of other subunits were observed after treatment with other extracts, with the only exception of NR1 subunit at a concentration of 500 mg/kg of the Borago methanolic extract (^*^p < 0.05) (Figure 5A). This result seems to be in agreement with other experimental evidences that reported the involvement of glutamatergic system in the ulcerative process. Indeed, some research studies ascribed the activation of such system to a physiologic protection event of myenteric structures following a central mechanism-induced stimulation, such as stress (32). The NR2A and NR2B mRNA increase is compatible with the receptor up-regulation effect pharmacologically due to a signal inhibition.

It is therefore conceivable that extract treatment plays a pivotal role in the NMDA-mediated acetylcholine release (number of ulcers), but also in the modulation of histamine-mediated H+ ions release with a molecular mechanism that leads to a cAMP increase (ulcer index).

### Cell Viability

**Figure 6:**
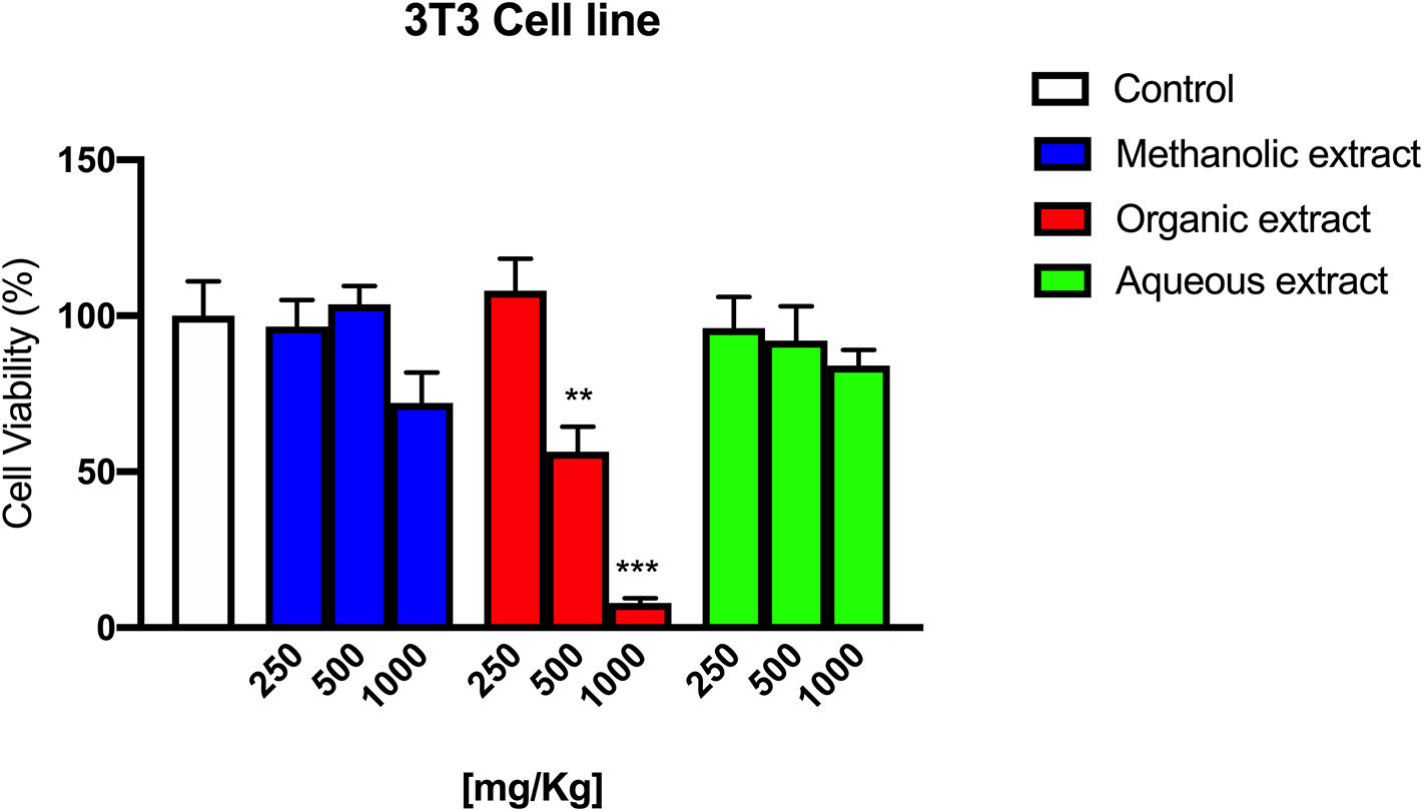
3T3 cells' viability following Borago methanolic, organic, and aqueous extracts exposure at different concentrations.

In Figure 6, the effect of different concentrations (250, 500, and 1,000 mg/kg) of Borago methanolic, organic, and aqueous extract on 3T3 cells is shown.

The Borago organic extract showed a significant cytotoxic effect at 500 and 1,000 mg/kg with a cell viability reduction of 43.6 % (^**^p < 0.01) and 92.1% (^***^p < 0.001), respectively, while methanolic and aqueous extracts exerted only a slight cytotoxic effect at 1,000 mg/kg.

## Discussion

The connection between the severe psychobiological and physiological stress and gastric damage has long been recognized (33).

It has been widely accepted that stomach wall secretion and motility alteration may manifest in stressful situations causing more or less severe ulcerations of gastrointestinal mucosa. This correlation was also observed in our animal model. Indeed, mucosa of stress-induced gastric ulcer rats showed a large number of ulcerative lesions, which disappeared when treated with omeprazole.

Recently, it has been reported that *Borago officinalis* extracts may have a gastroprotective effect against indomethacin-induced gastric ulcers (17). These effects seem to be due to the antioxidant properties of phenolic glycosides, flavonoids, and tannins present in the extracts. In our experimental stress-induced gastric ulcer model, the Borago extracts were able to significantly protect the gastric mucosa from the stress damage, confirming the finding of Gazwi and Mahmoud (17). In particular, we showed that all the extracts were able to decrease the severity and the number of stress ulcers. However, we found a different pattern of activity between the organic, methanolic, and aqueous extracts. Indeed, while the organic and methanolic extracts exerted their activity in a dose-dependent manner, the aqueous extract showed a significant activity only at the lowest doses tested. This latter finding could be related to a possible paradoxical effect, probably due to the presence of active hydrophilic components that might have high affinity toward a specific saturable receptor protein. Of course, more experiments are necessary in order to evaluate and confirm or contradict this speculation. As already mentioned, organic and methanolic extracts were able to protect the gastric mucosae at higher concentrations. Since Borago contains alkaloids such as theninine-4′-O-β-D-glucoside, which might be potentially dangerous to health, this could explain the toxicity elicited by high concentrations of organic extracts tested on 3T3 cell lines. However, the evidence that same doses of methanolic extract did not induce any cytotoxic effect, suggesting a different chemical composition of the two extracts, might make the methanolic and aqueous extracts as possible antiulcer agents. Moreover, the presence of Ca^2+^ antagonist in the Borago extract (18) suggests a possible role of Borago in the modulation of specific receptor complex. In this context, our results showed a modulation of Borago on the expression of NMDA receptor composition similar to that obtained with omeprazole. On the other hand, we found an increase in cAMP, which is itself an acid secretion stimulating factor (34). Therefore, high cAMP and low cGMP concentrations in the stomach might be related to the onset of ulcerative situations. It is known that NMDA receptor stimulation significantly reduced cAMP concentration while increasing that of cGMP, thus justifying the protective activity during the ulcerative lesion onset (35). Since we did not find a decrease in cAMP concentration, we believe that Borago extract activity should be addressed to NMDA receptor subunit modulation rather than a direct interaction with the receptor. Indeed, our data indicate an increase in cAMP elicited by the aqueous extract, thus excluding this pathway from the antiulcer properties of Borago extracts. The presence of the NMDA receptor on the enteric mucosa allowed us to hypothesize its involvement into inflammatory/toxic processes of the gastroenteric tract. In this context, some research showed conflicting results. NMDA glutamatergic receptors are linked, on one hand, to protective molecular mechanisms of gastric mucosa in an animal ulcer model and, on the other hand, to a glutamate-mediated inflammatory effect evidenced in intestinal myenteric neuron cultures. In our model of stress-induced ulcer, the expression of NMDA receptor subunits did not change, indicating that the receptor is not directly involved in the ulcer formation but rather a protective effect of the gastric mucosae as evidenced by the increase in NMDA subunit expression produced by the Borago extracts and omeprazole. Indeed, the animals subjected to stress and pretreated with AP5, a selective NMDA antagonist, had worse pathophysiological conditions of ulcers compared to those subjected to stress only.

## Conclusions

Results achieved in our experimentally induced stress rat model showed that Borago extracts have a strong anti-ulcer activity and highlight the presence of a number of active principles with different chemical–physical characteristics separated into the different extractive matrices. In particular, our results indicate that both methanolic and aqueous extracts possess a significant anti-ulcer effect without cytotoxic effects at all the concentrations tested. The fact that the aqueous solution was able to protect gastric mucosa only at the lowest doses tested could be addressed as a possible paradoxical phenomenon. Otherwise, the organic extract was found to be cytotoxic at the same concentrations where it was shown to exert anti-ulcer activity. Although the stress alone was unable to modify the expression of the NMDA receptor subunits, the Borago extracts and omeprazole, instead, showed an increase in NMDA receptor regulatory subunit expression, in particular as far as regarding the NR2B. Such observation might be compatible with the hypothesis of the protective effect exerted by the receptor on the gastric mucosa, suggesting a modulatory activity of Borago on NMDA glutamatergic receptor expression. Despite that this is a preliminary pilot study and further experiments are needed to understand the supramolecular mechanisms involved in the complex dynamics of stress-induced ulcer and the effective role of NMDA glutamatergic receptors, our data qualify Borago extracts (methanolic and aqueous) as potential protective agents. In addition, it is reasonable to ascribe such protective activity to a modulation of the NR2A and NR2B subunit expression. These findings are in agreement with the results by Golovynska et al. (36) and Tsai et al. (11), supporting the idea of a role of NMDA receptors in the protection of gastric mucosa.

## Data Availability Statement

All datasets generated for this study are included in the article/supplementary material.

## Ethics Statement

This animal study was reviewed and approved by Bioethical Committee of the Italian National Institute of Health.

## Author Contributions

AD, LC, MZ, and RA conceived and designed the experiments. AD, GC, RA, MZ, and LC performed all the experimental assay and statistical analysis. AD and LC wrote the manuscript. All authors contributed to the redaction, revision, and approval of the final manuscript.

## Conflict of Interest

The authors declare that the research was conducted in the absence of any commercial or financial relationships that could be construed as a potential conflict of interest.
